# Complementarity between Microbiome and Immunity May Account for the Potentiating Effect of Quercetin on the Antitumor Action of Cyclophosphamide in a Triple-Negative Breast Cancer Model

**DOI:** 10.3390/ph16101422

**Published:** 2023-10-06

**Authors:** Andrea Manni, Yuan-Wan Sun, Todd D. Schell, Tymofiy Lutsiv, Henry Thompson, Kun-Ming Chen, Cesar Aliaga, Junjia Zhu, Karam El-Bayoumy

**Affiliations:** 1 Penn State Health Milton S. Hershey Medical Center, Department of Medicine, Penn State College of Medicine, Hershey, PA 17033, USA; 2 Department of Biochemistry and Molecular Biology, Penn State College of Medicine, Hershey, PA 17033, USAkzc3@psu.edu (K.-M.C.); caliaga@pennstatehealth.psu.edu (C.A.); 3 Department of Microbiology and Immunology, Penn State College of Medicine, Hershey, PA 17033, USA; tschell@pennstatehealth.psu.edu; 4 Cancer Prevention Laboratory, Colorado State University, Fort Collins, CO 80523, USA; tymofiy.lutsiv@colostate.edu (T.L.); henry.thompson@colostate.edu (H.T.); 5 Department of Public Health Sciences, Penn State College of Medicine, Hershey, PA 17033, USA; jzhu2@pennstatehealth.psu.edu

**Keywords:** triple-negative breast cancer, microbiome, antitumor immunity, quercetin, Cyclophosphamide, 4T1 mammary tumor model

## Abstract

Immunotherapy targeting program cell death protein 1 (PD-1) in addition to chemotherapy has improved the survival of triple-negative breast cancer (TNBC) patients. However, the development of resistance and toxicity remain significant problems. Using the translationally relevant 4T1 mouse model of TNBC, we report here that dietary administration of the phytochemical quercetin enhanced the antitumor action of Cyclophosphamide, a cytotoxic drug with significant immunogenic effects that is part of the combination chemotherapy used in TNBC. We observed that quercetin favorably modified the host fecal microbiome by enriching species such as *Akkermansia muciniphilia,* which has been shown to improve response to anti-PD-1 therapy. We also show that quercetin and, to a greater extent, Cyclophosphamide increased the systemic frequency of T cells and NK cells. In addition, Cyclophosphamide alone and in combination with quercetin reduced the frequency of Treg, which is consistent with an antitumor immune response. On the other hand, Cyclophosphamide did not significantly alter the host microbiome, suggesting complementarity between microbiome- and immune-mediated mechanisms in potentiating the antitumor action of Cyclophosphamide by quercetin. Overall, these results support the potential for microbiota-centered dietary intervention to overcome resistance to chemoimmunotherapy in TNBC.

## 1. Introduction

Traditionally, cancer has been considered a cell-autonomous disease, and its treatment has focused on agents that target the neoplastic cells themselves. This theory has been replaced by the concept that tumor development and progression depend upon the interaction between cancer cells and multiple cell types surrounding the tumor, constituting the tumor microenvironment (TME) and systemic metabolic, inflammatory, and immune mechanisms [1,2]. The immune system has emerged as a significant factor in tumor control, which has been exploited therapeutically [3]. Cancer cells must escape immune surveillance to thrive, which can be accomplished systemically and at the target tissue level. Therefore, emphasis is placed on the development of immunogenic therapies aimed at reversing immune surveillance and activating an antitumor response [3]. Many conventionally used chemotherapeutic agents have indeed been shown to interact directly with immune cell subsets to stimulate antitumor immunity [4,5,6,7,8]. Immune checkpoint blockade with monoclonal antibodies targeting PD-1 is effective against multiple tumors, including TNBC [9,10,11,12]. Among breast cancer phenotypes, TNBC, associated with the worst prognosis [13,14,15], is the most immunogenic and amenable to immunotherapy [16,17,18]. The addition of immunotherapy to conventional chemotherapy in the neoadjuvant setting has increased the pathologic complete response (pCR) rate from 40% to 64.8% in women with early TNBC [19]. However, despite these positive results, the development of toxicity and resistance to chemotherapy and immunotherapy remains a significant challenge in cancer treatment [19].

Abundant evidence in the literature indicates that the gut microbiome plays a critical role in the antitumor effects of chemotherapy and immunotherapy in preclinical models and humans [20,21,22,23,24]. This finding supports the potential for microbiota-centered interventions to overcome resistance to immunogenic chemotherapy and immunotherapy [25]. Recent evidence indicates that nutritional interventions are promising, effective, and safe tools to favorably modify the microbiome leading to an increase in antitumor immune response [25,26].

Using the translationally relevant 4T1 in vivo mouse model of TNBC [27,28], we report here that dietary the administration of quercetin, a phytochemical with pleiotropic antitumor properties [29], enhances the antitumor action of Cyclophosphamide, a cytotoxic drug with significant immunogenic effects [4,5], which is part of the combination therapy used in TNBC [19]. Our data also provide novel information as the complementarity between the microbiome and immunity is mediating the antitumor effects of the combined administration of Cyclophosphamide and quercetin in an experimental model of TNBC.

## 2. Results

**Figure 1:**
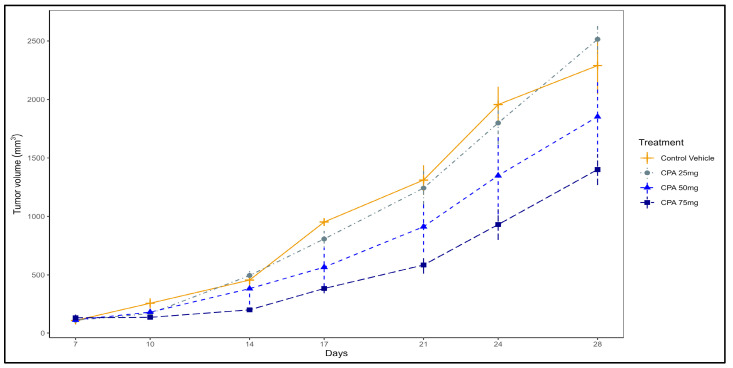
The effect of Cyclophosphamide at different doses on tumor volume using the 4T1 mouse model (n = 5 mice/group).

The effect of Cyclophosphamide at different doses on tumor volume using the 4T1 mouse model. The goal of this initial bioassay was to determine an effective but suboptimal dose of Cyclophosphamide to be used later in combination with quercetin to maximize our ability to detect possible additive/synergistic effects on the inhibition of tumor growth. As can be seen in Figure 1, among the doses tested, 75/mg/kg/BW exerted the strongest antitumor action, which was statistically significant (*p* < 0.05) starting at day 10 of treatment. On the other hand, the antitumor effect of 50 mg/kg/BW was more modest and not statistically significant. Hence, the 50 mg/kg/BW dose was selected for our subsequent experiments testing the combination treatment. No treatment-related toxicity was observed. Body weights and food intake were not different among groups, indicating no adverse effects of Cyclophosphamide. 

**Figure 2:**
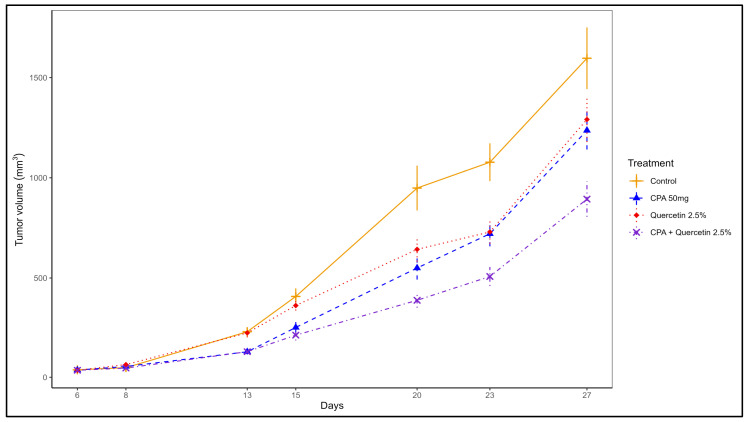
The effect of quercetin and Cyclophosphamide individually and in combination on tumor volume using the 4T1 mouse model (n = 10 mice/group).

Individual and combined effects of quercetin and Cyclophosphamide on tumor volume. Among the different dietary concentrations of quercetin tested, (1, 2.5, 5) the 2.5% was the most effective concentration in potentiating the antitumor action of Cyclophosphamide. The individual and combined effects of this dose of quercetin and Cyclophosphamide are shown in Figure 2. Statistical analysis using linear mixed effects models based on log transformed tumor volume shows that the combination exerted an additive antitumor effect compared to the individual treatments starting at day 20 (*p* < 0.05) (Figure 2). No statistically significant differences were detected in body weights and food intake among groups.

**Figure 3:**
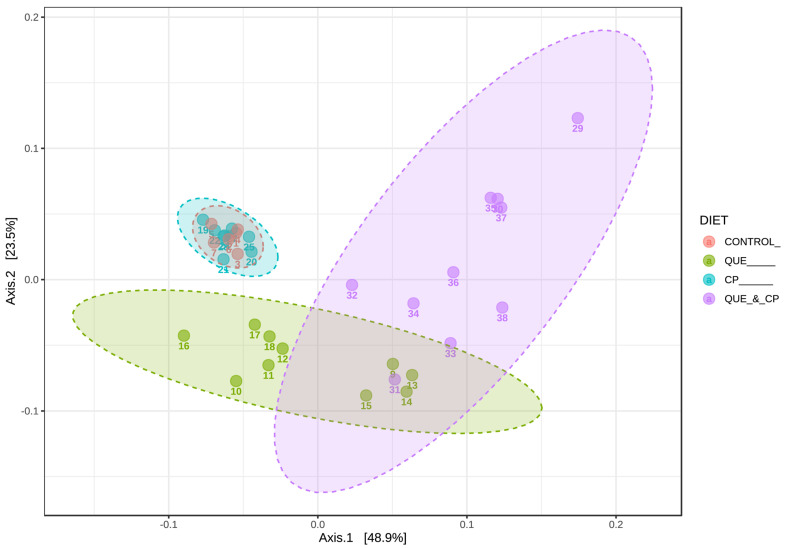
PCoA of β-diversity using weighted UniFrac distances. PERMANOVA testing: *pseudo-F* = 4.37, *p*-value < 0.001.

**Figure 4:**
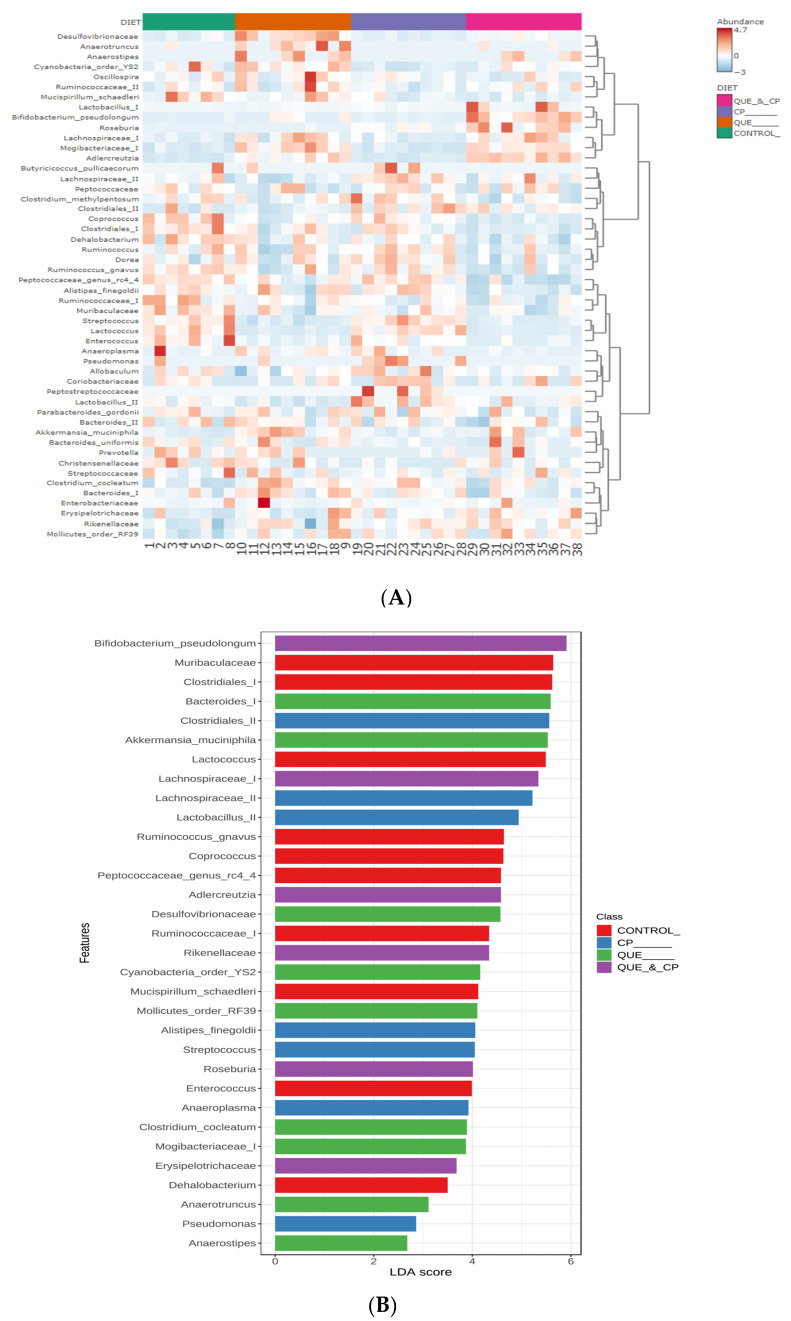
Differences in bacterial composition upon quercetin and cyclophosphamide administration. (**A**) Heatmap with hierarchical clustering analysis using Minkowski’s method and diet as a factor. (**B**) LEfSe analysis determined 28 bacterial taxa to explain the most-likely differences between the diet groups (FDR-corrected *p*-value < 0.05, LDA score > |2|).

Individual and combined effects of quercetin and Cyclophosphamide on host microbiome. Microbiome analysis of the fecal samples revealed that while α-diversity (i.e., within samples diversity) was not affected, β-diversity (i.e., between samples diversity) was significantly impacted (*p* < 0.001), showing distinct microbial communities within each treatment group. Quercetin induces a more dramatic separation of samples from the control than Cyclophosphamide in PCoA, additionally enhancing Cyclophosphamide-driven differences in their bacterial composition (Figure 3). Bacterial patterns underlying such differences can be visualized in a heatmap (Figure 4), where bacteria on the bottom of the list seem to be the reason to draw parallels between quercetin-containing diet groups. In contrast, the middle section of bacteria combines control and Cyclophosphamide-treated groups. 

Differential abundance analysis revealed that quercetin significantly induces abundant representatives of *Anaerotruncus*, *Mogibacteriaceae*, *Clostridium cocleatum*, RF39 (*Mollicutes*), *Adlercreutzia*, *Desulfovibrionaceae, Lachnospiraceae*, and especially *Akkermansia muciniphila* compared to the control. In combination with Cyclophosphamide, it induces presence of *Erysipelotrichaceae*, *Mogibacteriaceae*, *Roseburia*, RF39 (*Mollicutes*), *Rikenellaceae*, *Adlercreutzia*, *Akkermansia muciniphila*, *Lachnospiraceae*, and *Bifidobacterium pseudolongum*. 

Overall, quercetin favorably modifies the microbiome with enrichments of species such as *Akkermansia muciniphilia*, which has been shown to improve response to anti-PD1 therapy [23,30], thus emphasizing the link between the microbiome and immune cell regulation.

**Figure 5:**
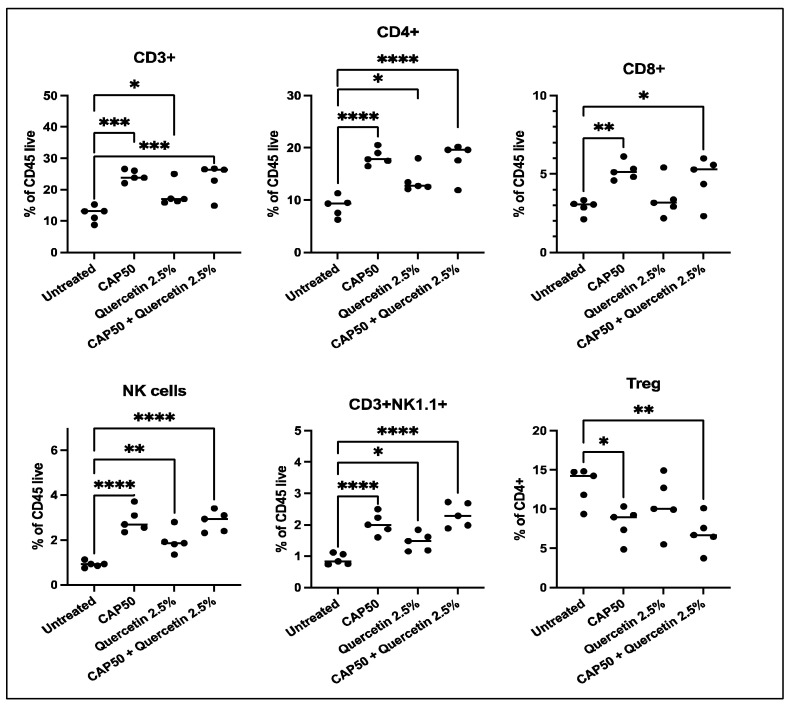
Systemic levels of T cells and NK cells are increased in quercetin- and cyclophosphamide-treated mice. Flow cytometric analysis was used to assess the frequency of lymphocyte populations in the spleens of 4T1-tumor-bearing mice; each dot represents the results from one mouse. n = 5 per group. Statistical significance versus untreated mice is indicated; * *p* < 0.05, ** *p* < 0.01, *** *p* < 0.001, **** *p* < 0.0001.

Individual and combined effects of quercetin and Cyclophosphamide on host immunity. Flow cytometric analysis of tumor bearing mice splenocytes revealed that both quercetin and Cyclophosphamide increased the frequency of total CD3+ cells and NK cells, although this increase was more dramatic with Cyclophosphamide (Figure 5). A similar increase was observed in CD3+NK1.1+ cells, a subset that includes natural killer T cells. This increase in total T cells was mainly driven by an increase in the CD4 T cell frequency in all treatment groups and by a slight increase in CD8 T cells of mice treated with Cyclophosphamide. Notably, despite the overall increase in CD4+ T cells, Cyclophosphamide treatment significantly reduced the CD4+FoxP3+CD25+ regulatory T cell population alone and in combination with quercetin. These results indicate that Cyclophosphamide and quercetin enhanced the systemic frequency of T cells and NK cells while reducing that of Treg cells, suggesting that these critical antitumor immune cells can be mobilized by combination therapy.

## 3. Discussion

TNBC, the breast cancer phenotype associated with the worst prognosis [13,14,15], does not benefit from molecularly targeted therapy, such as hormone treatment or anti-HER-2neu therapy, because these tumors lack the expression of estrogen and progesterone receptors and epidermal growth factor receptor 2 (HER-2neu). Therefore, systemic chemotherapy has traditionally been the only treatment option for these patients. However, the transition of our vision of cancer from a cell-autonomous disease to an ecological disorder conditioned by the interaction between the tumor cells and local and systemic, metabolic, inflammatory, and immune mechanisms introduced the opportunity to develop a targeted therapy for TNBC. While breast cancer has been traditionally viewed as immunologically silent, TNBC is characterized by higher levels of tumor-infiltrating lymphocytes (TILS) [16] which have been shown to have both therapeutic and prognostic value [31]. Furthermore, the increased expression of PD-L1 in both tumor and immune cells [17,18] suggested that this aggressive breast cancer phenotype may be responsive to immune checkpoint inhibitor (ICI) therapy. The addition of anti-PD1 therapy with pembrolizumab to combination chemotherapy has indeed improved overall survival in TNBC patients with both early and advanced disease [19,32]. However, the development of resistance and toxicity remain significant medical problems. 

Using the translationally relevant 4T1 syngeneic mouse model of TNBC [27,28], these experiments were designed to test the hypothesis that the phytochemical quercetin increases the efficacy of chemotherapy. Phytochemicals are natural plant-derived compounds found in fruits and vegetables with a wide range of antitumor properties [33]. Since tumor growth and progression is mediated by the activation of multiple overlapping signaling pathways, the pleotropic antitumor action of phytochemicals may offer an advantage over the use of synthetic drugs which typically affect a single pathway. The use of these drugs while initially effective, frequently results in drug resistance due to the compensatory activation of alternative pathways. Consequently, the sequential or combined use of several inhibitors is usually employed and frequently associated with excessive toxicity and high costs [34,35]. When compared to synthetic molecules, phytochemicals are widely available, highly tolerated, and cost-effective [36]. Among them, quercetin is most effective against TNBC and enhances cytotoxic drug antitumor activity in various experimental cancer models [29]. Most relevant to our studies, quercetin has been shown to potentiate the anti-tumor action of Adriamycin-induced cardiotoxicity [37]. This finding is in line with the protective effects of phytochemicals against chemotherapy-induced toxicity, as also observed in other systems [38,39,40,41]. Overall, most of the studies have been conducted in vitro, thus limiting the translational significance of their findings as they do not consider critical variables such as the tumor ecosystem and in vivo bioavailability issues. We observed that the dietary administration of quercetin significantly increased the antitumor action of Cyclophosphamide (Figure 2), a cytotoxic drug that, like Adriamycin, is part of the combination chemotherapy of TNBC and whose mechanism of antitumor action involves the upregulation of the antitumor immune response [4,8,42].

To gain insight into the mechanisms of antitumor actions of Cyclophosphamide and quercetin individually and in combination, we evaluated the effects of our treatments on the host microbiome and immune phenotype. The overall favorable influence of a quercetin-containing diet on the host microbiome (Figure 4) is in line with recent evidence pointing to nutritional interventions as practical tools to create an antitumor environment through the microbiome-induced enhancement of antitumor immunity [25,26]. Quercetin significantly increased immune biomarkers consistent with a tumor protective phenotype (Figure 5). However, the causal relationship between the microbiome and immune changes needs to be directly tested in future experiments using germ-free mice. A novel observation of our investigations is that quercetin enriched the presence of *Akkermansia muciniphila* (Figure 5), a species present in the human gut microbiota [43,44] that has been shown to improve response to anti-PD1 therapy in mice and humans [23,30]. This finding is relevant to treating patients with TNBC since anti-PD1 therapy with pembrolizumab is a critical component of its combination treatment. On the other hand, Cyclophosphamide administration did not significantly influence the host microbiome (Figure 3 and Figure 4), but rather caused a significant increase in the systemic frequency of T cells and NK cells, and a significant decrease in that of Treg (Figure 5), with both findings being consistent with an antitumor immune response. Prior studies have shown that Cyclophosphamide can reduce Tregs in end-stage cancer patients [45] and the 4T1 breast cancer model [46]. Cyclophosphamide at low doses (5 mg/kg) was found to increase circulating and 4T1 tumor-infiltrating NK cells while higher doses provided ad libitum reduced NK cell numbers. Our results suggest that limited Cyclophosphomide dosing at 50 mg/kg was sufficient to increase both systemic T cell and NK cell frequencies while also suppressing Treg cell accumulation. A low abundance of Treg has been proposed as a predictive biomarker of pathological complete response after neoadjuvant chemotherapy in TNBC [47]. Thus, the increased ratio or T cell and NK cells to Tregs may contribute toward the anti-tumor efficacy observed in this study. Recently, the accumulation of immature immunoregulatory NK cells in TNBC has been associated with tumor progression [48], so it will be important to understand how the phenotype of NK cells and T cells in TNBC may be changed by Cyclophosphamide, quercetin, and their combination. In the aggregate, these results indicate that the superior antitumor effect of the combination of quercetin and Cyclophosphamide (Figure 2) is due to different but complementary mechanisms of action.

## 4. Materials and Methods

Experimental System. In these experiments, we used the 4T1 mouse model, a syngeneic in vivo model of TNBC, which has been successfully used in our laboratories [27] and others [28]. Briefly, suspended early-passage 4T1 cells (5 × 10^3^) in 100 µL of phosphate-buffered saline (PBS) were injected into one axillary mammary fat pad per mouse of 5–6-week-old female immunocompetent BALB/c mice (Tacomic Biosciences, Inc., Rensselaer, NY USA). Mice were kept in a pathogen-free animal facility and housed at standard conditions (12 h light/12 h dark cycles, 50% humidity, 23 ± 1 °C) and allowed to acclimatize for one week before injecting 4T1 cells. A Vernier caliper was used to measure tumor growth twice a week, and tumor volumes were calculated using the formula V(mm^3^) = L (central axis) × W (minor axis)/2. The mice had access to water and food (AIN93M) ad. Lib.; body weights and food consumption were measured twice a week during the progress of the bioassay. Our animal studies were conducted according to the National Institutes of Health, USA, prior to their initiation. The Penn State College of Medicine Institutional Animal Care and Use Committee (IACUC Approval Date: 30 October 2020) reviewed and approved all experiments (USDA Registration Number: 23-R-0021).

Cyclophosphamide dose response study. Before testing the potentiating effect of quercetin on Cyclophosphamide, we needed to determine an optimal dose of Cyclophosphamide that exerts antitumor action without causing toxicity. Four groups of mice (n = 5/group) injected with 4T1 cells one week before they were randomly assigned to receive i.p. control vehicle, 25, 50, or 75 mg/Kg BW of Cyclophosphamide. After the tumor cells injection, a second drug dose was administered on day 21. Tumor volume and body weight were monitored as described above. Although Cyclophosphamide has not been used in the 4T1 mouse model, these doses were comparable to those reported in the literature in other mouse models [49].

Individual and combined effects of quercetin and Cyclophosphamide. We selected a dose of Cyclophosphamide of 50 mg/kg BW. While effective, this dose was suboptimal (see Results), thus allowing us to test the potential additive/synergistic effects of adding dietary quercetin. We used quercetin at 1, 2.5, and 5% in the diet since the literature data documented that mice tolerated these dietary levels [29]. Hence, eight separate groups of mice (n = 10/group) were injected with 4T1 cells one week before they were randomly assigned to receive the following: (1) control vehicle; (2) Cyclophosphamide 50 mg kg i.p. (on day 7 and 21 post-tumor cell injection); (3) quercetin 1%; (4) quercetin 2.5%; (5) quercetin 5%; (6) quercetin 1% plus Cyclophosphamide; (7) quercetin 2.5% plus Cyclophosphamide; and (8) quercetin 5% plus Cyclophosphamide. Diets containing quercetin were prepared every week, packed in plastic zipper sealed bags, flushed with nitrogen, and kept at four °C until use. Fresh diets were supplied every other day. Tumor volumes and body weights were monitored, as described above. At termination, fecal samples and spleens were obtained, processed, and stored for microbiome analysis and immune phenotyping.

Microbiome analysis. Microbiome analysis of fecal specimens was performed, as recently published by us [50]. Briefly, DNA was extracted from fecal pellets using the QiaAMP DNA Stool Mini Kit (Qiagen, Germantown, MD, USA). 2 × 300 bp paired-end sequencing libraries of the V4 region of the 16S rRNA gene were constructed by following the Schloss MiSeq Wet Lab SOP, followed by sequencing on an Illumina MiSeq. All 16S rRNA gene sequences were processed with Qimme2 (version 2022.2) bioinformatics tool.

Immune studies. The determination of the frequency of lymphocyte populations in the spleens of mice from the different treatment groups was performed by flow cytometry, as published by us [51]. Spleens were harvested at day 27 post treatment initiation and processed to single cells, as previously described [52]. Red-blood-cell-depleted cells were stained with the following antibodies from BD Biosciences: anti-CD45.2-BV480, anti-CD3-PE, anti-CD8a-BV786, anti-CD4-BB700, and anti-NK1.1-BV421, or alternatively withanti-FoxP3-BV421 and anti-CD25-APC. Dead cells were excluded using FVS780. Samples were run on a BD Biosciences FACSymphony A3, and data were analyzed using FlowJo software (v. 10.9). 

Statistical Analysis of tumor growth. The linear mixed-effect model for repeated measures was used to analyze the tumor growth data (results related to Figure 1 and Figure 2). The outcome variable tumor volume was log-transformed to satisfy the underlying statistical assumptions. Within each time point, group effect was examined, and the pair-wise comparisons between groups were made. To study the interaction effects between quercetin and Cyclophosphamide, we used two-way ANOVA models at each time point (results related to Figure 2). All analyses were performed using statistical software SAS version 9.4 (SAS Institute Inc., Cary, NC, USA). All tests were two-sided, and the statistical significance level used was 0.05. Due to the exploratory nature of this study, the *p*-values were not adjusted for multiple comparisons. 

Microbiome analysis. Sequence data on the V4 region of the 16S rRNA gene were processed in QIIME2 (version 2022.2; [53]) using the DADA2 pipeline [54]. The resulting ASVs tables were analyzed in QIIME2 and MicrobiomeAnalyst web-platform [55,56]. In particular, diversity analysis included observed features, Pielou’s evenness, Faith’s phylogenetic diversity, and Shannon’s diversity index metrics of α-diversity tested with Kruskal–Wallis non-parametric test, as well as principal coordinates analysis (PCoA) using Jaccard, Bray–Curtis, and phylogenic weighted and unweighted UniFrac distances with permutational multivariate analysis of variance (PERMANOVA) to assess β-diversity. Ward’s hierarchical clustering algorithm based on Minkowski distances was visualized on a heatmap to determine batters of bacterial changes across samples. The linear discriminant analysis (LDA) effect size (LEfSe) method [57] was used to perform differential abundance analysis and identify bacterial biomarkers of the treatment groups using FDR-adjusted *p*-value < 0.05 and logarithmic LDA score absolute value > 2.0 as cutoffs. 

Immune Cell Analysis. Statistical differences in the means of immune cell frequencies were evaluated by ordinary one-way ANOVA with multiple comparisons using Prism (v. 10.0.1). 

## 5. Conclusions

Our results support the complementarity between microbiome and immune-mediated mechanisms in potentiating the antitumor action of Cyclophosphamide by quercetin in a well-defined animal model of TNBC.

## Data Availability

Data not provided in this manuscript is available upon request to the corresponding authors.
